# Genotype Imputation to Improve the Cost-Efficiency of Genomic Selection in Rabbits

**DOI:** 10.3390/ani11030803

**Published:** 2021-03-13

**Authors:** Enrico Mancin, Bolívar Samuel Sosa-Madrid, Agustín Blasco, Noelia Ibáñez-Escriche

**Affiliations:** 1 Department of Agronomy, Food, Natural Resources, Animals and Environment (DAFNAE), University of Padova, viale dell’Università 16, 35020 Legnaro, PD, Italy; enrico.mancin@phd.unipd.it; 2 Institute for Animal Science and Technology, Universitat Politècnica de València, 46022 Valencia, Spain; ablasco@dca.upv.es

**Keywords:** genomic selection, imputation, litter size, rabbits, genomic simulation

## Abstract

**Simple Summary:**

Genotyping costs are still the major limitation for the uptake of genomic selection by the rabbit meat industry, as a large number of genetic markers are needed for improving the prediction of breeding values by genomic data. In this study, several genotyping strategies were examined through simulation scenarios to disentangle the best feasible options of implementing genomic selection in rabbit breeding programs. Most scenarios emphasized the genotyping of candidate animals with a low Single Nucleotide Polymorphism (SNP) density platform. Imputation accuracies were high for the scenarios with ancestors genotyped at high or medium SNP-densities. However, the scenario with male ancestors genotyped at high SNP-density and only dams genotyped at medium SNP-density showed the best economically feasible strategy, taking into account the trade-off among genotyping costs, the accuracy of breeding values and response to selection. The results confirmed that by combining the imputation technique with a mindful selection of the animals to be genotyped, it is possible to achieve better performance than Best Linear Unbiased Prediction (BLUP), reducing genotyping cost at the same time.

**Abstract:**

Genomic selection uses genetic marker information to predict genomic breeding values (gEBVs), and can be a suitable tool for selecting low-hereditability traits such as litter size in rabbits. However, genotyping costs in rabbits are still too high to enable genomic prediction in selective breeding programs. One method for decreasing genotyping costs is the genotype imputation, where parents are genotyped at high SNP-density (HD) and the progeny are genotyped at lower SNP-density, followed by imputation to HD. The aim of this study was to disentangle the best imputation strategies with a trade-off between genotyping costs and the accuracy of breeding values for litter size. A selection process, mimicking a commercial breeding rabbit selection program for litter size, was simulated. Two different Quantitative Trait Nucleotide (QTN) models (QTN_5 and QTN_44) were generated 36 times each. From these simulations, seven different scenarios (S1–S7) and a further replicate of the third scenario (S3_A) were created. Scenarios consist of a different combination of genotyping strategies. In these scenarios, ancestors and progeny were genotyped with a mix of three different platforms, containing 200,000, 60,000, and 600 SNPs under a cost of EUR 100, 50 and 11 per animal, respectively. Imputation accuracy (IA) was measured as a Pearson’s correlation between true genotype and imputed genotype, whilst the accuracy of gEBVs was the correlation between true breeding value and the estimated one. The relationships between IA, the accuracy of gEBVs, genotyping costs, and response to selection were examined under each QTN model. QTN_44 presented better performance, according to the results of genomic prediction, but the same ranks between scenarios remained in both QTN models. The highest IA (0.99) and the accuracy of gEBVs (0.26; QTN_44, and 0.228; QTN_5) were observed in S1 where all ancestors were genotyped at HD and progeny at medium SNP-density (MD). Nevertheless, this was the most expensive scenario compared to the others in which the progenies were genotyped at low SNP-density (LD). Scenarios with low average costs presented low IA, particularly when female ancestors were genotyped at LD (S5) or non-genotyped (S7). The S3_A, imputing whole-genomes, had the lowest accuracy of gEBVs (0.09), even worse than Best Linear Unbiased Prediction (BLUP). The best trade-off between genotyping costs and the accuracy of gEBVs (0.234; QTN_44 and 0.199) was in S6, in which dams were genotyped with MD whilst grand-dams were non-genotyped. However, this relationship would depend mainly on the distribution of QTN and SNP across the genome, suggesting further studies on the characterization of the rabbit genome in the Spanish lines. In summary, genomic selection with genotype imputation is feasible in the rabbit industry, considering only genotyping strategies with suitable IA, accuracy of gEBVs, genotyping costs, and response to selection.

## 1. Introduction

The rabbit industry still plays an important role throughout the agricultural sector in some European countries, such as Spain, Italy, France, Hungary, Portugal, Germany, Belgium, Poland and Malta [1,2]. In recent years, the rabbit industry is currently facing a critical period, mainly due to the increase in feeding, management costs and a constant decline in rabbit meat consumption. Hence, farmers and researchers have been looking for promising strategies to improve the current situation, one of them being the optimization of genetic selection by using genomic information. Genetic selection can improve productive and reproductive traits, such as meat characteristics, reducing feeding and management costs, which makes rabbit more appealing for consumers, and hence, will aid the rabbit industry [1]. Reproductive traits, especially litter size, are those with relevant economic weight in the rabbit industry [3]. However, the response to selection for this trait has been relatively low using traditional selection by Best Linear Unbiased Prediction (BLUP), mainly due to its low-heritability [4,5,6]. Selection using genomic information can be an efficient tool to guarantee a higher genetic gain for traits with low heritability and measured in only one sex such as litter size. [7,8]. Genomic selection has generally showed better accuracy for the predicted breeding value (BV) [9] due to a potentially more accurate kindship estimation between animals. This method has produced positive selection results for traits in dairy cattle [10,11], poultry [12] and pigs [13,14,15]. However, in rabbits, a high-density commercial Single Nucleotide Polymorphism (SNP) platform (~200K SNPs) was not available until 2015, which has delayed genomic selection application [16]. Further, additional issues such as the small economic value of paternal rabbits, the costs of the commercial SNP platform, and the short generation interval are still limiting genomic selection as an evaluating method [17]. Strategies allowing us to diminish high genotyping costs are vital in the rabbit industry. The imputation of a low SNP-density platform using a high SNP-density platform has been carried out in other species, keeping or improving the genetic progress. The technique normally consists of genotyping ancestors (grandparents and parents) at high SNP-density in order to assign (impute) the most likely SNP allele to missing genotypes of young candidate animals genotyped at lower SNP-density [10,18,19]. This approach depends on multiple factors concerning the level of SNP density, methods, the structure and size of the reference population, the minor allele frequency of missing or untyped SNPs, the genetic architecture of traits and the particular breeding scheme of a livestock species [20,21,22].

The aim of this study was to disentangle the most appropriate imputation strategies for implementing genomic selection in maternal rabbit breeding schemes. Under this aim, the imputation accuracy was evaluated from low to moderate SNP-density platforms considering the cost-effectiveness of each strategy. In addition, we investigated how the imputation strategies influence the estimation of breeding values and consequently the response to selection.

## 2. Materials and Methods

### 2.1. Simulation Structure

**Figure 1:**
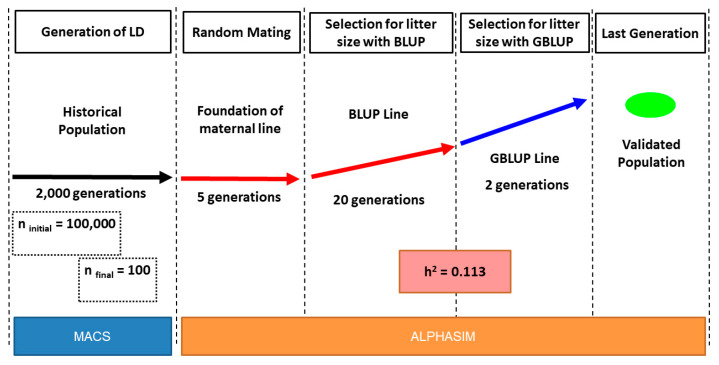
Design of simulations. A first period, generating the linkage disequilibrium (LD) across the rabbit genome using *MaCS* program. The remaining periods are carried out using *AlphaSim*: foundation of maternal line, selection for litter size with Best Linear Unbiased Prediction (BLUP), and with Genomic Best Linear Unbiased Prediction (GBLUP).

We used stochastic simulation to create the populations for developing imputation analyses. The structure of simulations is shown in Figure 1. The simulations were performed by the AlphaSim program [23], available at https://alphagenes.roslin.ed.ac.uk/wp/software-2/alphasimr/ (accessed on 10 June 2020). The objective was to simulate a rabbit population mimicking a single maternal line under the common rabbit breeding scheme of a small nucleus breeding. The simulated trait was litter size at birth. First, founder genomes were generated. The rabbit genome was simulated by sampling 2000 haplotype sequences for each of 20 chromosomes using the Markovian Coalescent Simulator (*MaCS*) [24]. Each chromosome was 100 cM long, as genetic distance, and included 124.43 × 10^6^ base pairs, as physical distance. Chromosomes were simulated according to rabbit population history defined by the *MaCS* program using a per-site recombination rate 8.57 × 10^−9^, a per-site mutation rate 1.74 × 10^−9^, and an effective population size varying over time (according to “Internal rabbit” as an option of population history in *MaCS*). Later, in the first generation of the foundation of the maternal line (initial population), quantitative trait nucleotides (QTNs) were chosen randomly from the segregating sequence variants and an equal number of QTNs were assigned to each chromosome. The QTNs had additive effects sampled from a Gamma distribution with a shape of 0.60 and a scale of 0.80. These parameters were chosen after exploratory analysis evaluating various values of Gamma distribution parameters against selection response after 20 generations (analyses not shown). The heritability and residual variance were calculated relative to the additive variance in the initial population. The heritability was 0.113 and the trait genetic variance was 0.675 in the base population [5,25].

After five generations of random mating, for details as in [26], a base population was established using 138 does and 77 sires. The selection was carried out to select 70 does and 35 sires per generation, involving 20 generations with the BLUP method (see the parameters in Table S1). Litter size was assumed as a trait of sex-limited expression, hence, only does presented phenotypic records. In every generation, the 70 does from the previous generation with the highest estimated breeding values (EBVs) were selected to produce the next generation. A total of 35 males were also selected, which stand for 25 principal and 10 surrogate sires used in practice. In addition, two further generations evaluated with the Genomic Best Linear Unbiased Prediction (GBLUP) method were generated to obtain the last population, which consisted of a progeny with 1500 does and 1500 males. The two selection methods used in these simulations (BLUP and GBLUP) are directly implemented in the *AlphaSim* program.

The high SNP-density platform (HD; 200,000 SNPs) used in genomic selection was generated by AlphaSim. Another two SNP platforms were set up according to SNP-density size: medium SNP-density (MD; 6000 SNPs) and low SNP-density (LD; 600 SNPs) platforms. All SNPs were assigned proportionally per chromosome. Genetic markers for the MD platform were selected at random from the HD platform and, consequently, genetic markers for the LD platform were selected at random from the MD platform (see the parameters in Table S1). Regarding costs for implementing genomic selection, we only considered the costs of genotyping based on the SNP platforms in the last three generations; therefore, other indirect costs were ignored. Each platform contains 96 “BeadChips” (genotyping proof). We assumed that the cost of the HD platform is EUR 9600. For the other platforms, the costs are around EUR 4800 and EUR 1056 for MD and LD, respectively. This information came from Thermo Fisher Scientific Inc. supplied by genotyping budgets for previous genomic experiments in the Animal Breeding Group at the Institute for Animal Science and Technology in the Universitat Politècnica de València.

An underlying polygenic nature was assumed for litter size, representing the genetic architecture of complex traits [27]. We considered two QTN models, depicting: (1) a polygenic trait controlled by many QTNs with a total of 880 (44 per chromosome; QTN_44) and (2) a polygenic trait controlled by a small number of 100 QTNs (5 per chromosome; QTN_5). Simulated data were generated from 36 replicates for every QTN model. The results were summarized over 36 replicates within the QTN model, and presented graphically. The graphics were obtained by the R program [28].

### 2.2. Imputation Strategies

All imputation analyses were developed by AlphaImpute program *v1.96*. This program is available at https://alphagenes.roslin.ed.ac.uk/wp/software-2/alphaimpute/ (accessed on 15 August 2020). AlphaImpute uses a hybrid imputation algorithm in which the first step consists of a long-range phasing process, followed up haplotypes library construction, and finally, pedigree-based imputations are performed [29].

Imputation accuracy (IA) was measured by the Pearson’s correlation between the imputed allele and the true genotype at untyped SNP markers. The correlation was computed one individual at a time and averaged over individuals [22]. This parameter stands for the genotype probability for reasons sketched out in [30]. Genotype yield was also computed for each imputation strategy below, as the percentage of the SNP allele calls at untyped SNP markers after the imputation process.

To assess the trade-off between IA, genomic prediction accuracy and genotyping cost, a number of hypothetical test scenarios were established, as outlined below and represented in Table 1. We analyzed the simulated data in seven sets of hypothetical scenarios with a replicate of the third scenario (S3_A). All grand-sires and sires were genotyped at HD platforms, whilst grand-dams and dams were genotyped according to the imputation strategy. The progenies were genotyped at the LD platform except the first scenario (S1), as this scenario used MD platforms to genotype progeny and HD platforms for the genotyping of grand-dams and dams. The second scenario (S2) was like the first scenario, but LD platforms were used for progeny genotyping. The third scenario (S3) used MD platforms for the genotyping of grand-dams, and only half of the progeny was genotyped. S3_A included the further half of the progeny, having their imputed whole-genomes. The fourth scenario (S4) used MD platforms for the genotyping of grand-dams and dams, whilst the fifth scenario (S5) used LD platforms. The sixth scenario (S6) used MD platforms for genotyping dams, but the grand-dams were non-genotyped. The seventh scenario (S7) had non-genotyped grand-dams and dams. These above-mentioned scenarios summarized all exploratory analyses concerning imputation strategies.

### 2.3. Estimating Breeding Values and Response to Genomic Selection

Typically, genomic selection entails a training population or reference population (with genotyped and phenotyped individuals) and an evaluated population of young candidates (with only genotyped individuals), using pseudo-phenotypes for sex-limited traits [7,31]. In this study, the rabbit reference population comprised up to 300 females (Table 2). As the reference population was small, genomic prediction was estimated using the Single-Step GBLUP (ssGBLUP) algorithm. It allows us to evaluate jointly non-genotyped and genotyped animals, combining pedigree and markers information into one matrix [32,33]. Otherwise, prediction by GBLUP hinders a greater rate of genetic progress compared to BLUP selection, with numerically small reference populations [11,34].

In each simulation, the accuracy of genomic breeding values (gEBVs) was estimated using the imputed genotypes of the eight scenarios described above. In addition, the EBVs of candidate animals were also estimated using only pedigree information (BLUP scenarios). The model was the same for BLUP and ssGBLUP:

(1) y=1μ+Za+e

where y is the vector of phenotypes, μ is the population’s mean, a is the vector of additive genetic effects of animals, e is the vector of residuals, and Z is the incidence matrix for additive genetic effects. Residual effects are sampled from distribution *N*(**0**,***I***$\sigma_{e}^{2}$). In BLUP, random additive genetic effects are sampled from distribution *N*(**0**,***A***$\sigma_{a}^{2}$); $\sigma_{a}^{2}$ is the genetic additive variance and ***A*** is the identity by descent (IBD) relationship matrix constructed from pedigree information. In ssGBLUP, additive genetic effects are sampled from distribution with ***a***∼*N*(**0**,***H***$\sigma_{a}^{2}$). ***H*** matrix can be interpreted as the (co)variances of multivariate normal distributions of additive effects of both genotyped and non-genotyped animals [32,33]. In ssGBLUP, the inverse of the (co)variance structure of random effect was replaced by $H^{-1}$, described as:

(2) $$H^{-1}=A^{-1}+\left(\begin{array}{cc} 0 & 0\\ 0 & G^{-1}-A_{22}^{-1} \end{array}\right)$$

where $A^{-1}$ is the inverse of pedigree matrix and $A_{22}^{-1}$ is the inverse of the sub-covariance structure containing only genotyped animals. $G^{-1}$ is the inverse of the matrix built as described in [35]. According to Garcia-Baccino et al. [36] and Aguilar et al. [37], $A^{-1}$ was computed accounting for inbreeding in order to avoid inflation (bias), particularly for BLUP scenarios, and to minimize blending problems between genomic and pedigree matrices.

All analyses were performed using the *BLUPF90* suite of programs under their standard parameters of quality control for genomic database [38]. Pedigree information from the 23rd to 28th generations was retained (Table 2). Phenotypic data comprised does from the 23rd to 27th generations. Phenotypic records of progenies at the last generation (28th generation) were not considered because they represented young candidate animals. Although all scenarios (S1–S7) used genotypes of grand-sires and sires (26th and 27th generations) for genomic prediction, a few scenarios (S1 and S2) also used all genotypes of grand-dam and dams. In the other scenarios (S3–S7), genotypes from grand-dams and dams were discarded due to low IA and the large number of missing SNPs [39]. On the other hand, in S3, EBVs of non-genotyped progeny were estimated as means of their parents’ EBVs (Table 1).

The accuracy of gEBVs was measured by the Pearson’s correlation between predicted and true breeding values (TBVs) on the progeny at the validated population, animals belonging to the 28th generation. To assess the gain of accuracies when genomic information was introduced, the mathematical differences of accuracies between each scenario (S1–S7) and BLUP were calculated within each simulation.

This study emphasizes the genetic improvement via doe selection. Hence, response to selection was calculated by subtracting the TBV mean of young candidates (1500 does), within each imputation strategy, from the TBV mean of the 150 selected individuals. We also computed the percentage of candidate animals correctly selected. Results within each scenario are exposed, comparing IA, the accuracy of gEBVs, the selection response and genotyping cost.

## 3. Results

### 3.1. Simulation Outcomes

**Figure 2:**
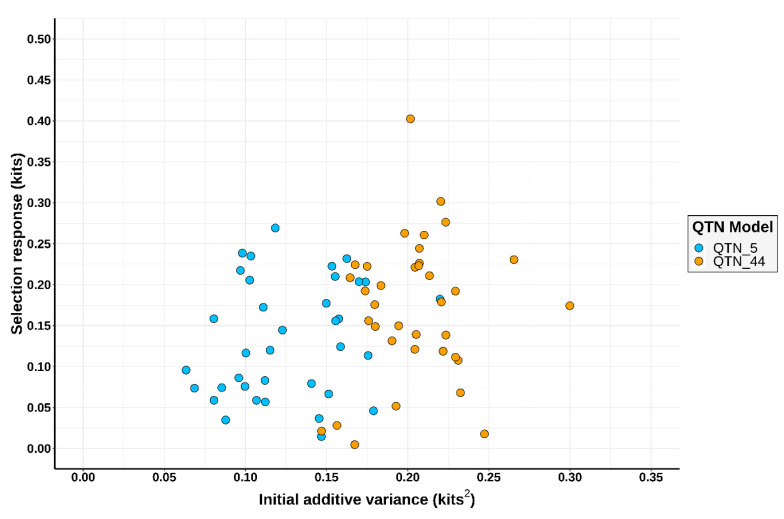
Relationship between initial additive variance and the response to genomic selection. The initial additive variance is the additive genetic variance at the 25th generation in which the genomic evaluations begin. QTN_5 stands for the QTN model with 5 QTNs per chromosome and QTN_44 stands for the QTN model with 44 QTNs per chromosome. The simulated rabbit genome comprised 20 chromosomes.

The outcomes from the 36 simulations were similar with regards to the response to the selection of experiments using Spanish rabbit lines and the BLUP method for genetic evaluations: an average of 2.53 kits after 20 generations of selection [4,5]. For genomic selection, the average response to selection across QTN models computed for the first two generations with HD platforms was 0.15 kits (0.13 and 0.17 for QTN_5 and QTN_44 models, respectively), which corresponds to a response per generation of 0.08 (ranging between 0.002 and 0.20) across QTN models. These results are in line with outcomes of pig genomic selection experiments [40], as hitherto there have been no empirical genomic selection experiments in rabbits. As expected, the additive genetic variance was smaller in QTN_5 than in QTN_44. As expected, scenarios with 44 QTNs per chromosome presented higher additive genetic variance than QTN_5 ones. This is strictly correlated with the number of QTNs that were fixed during haplotype creation. On the other hand, this discrepancy did not have a clear effect on the response to selection as reported on Figure 2.

### 3.2. Performance of Imputation Strategies

**Figure 3:**
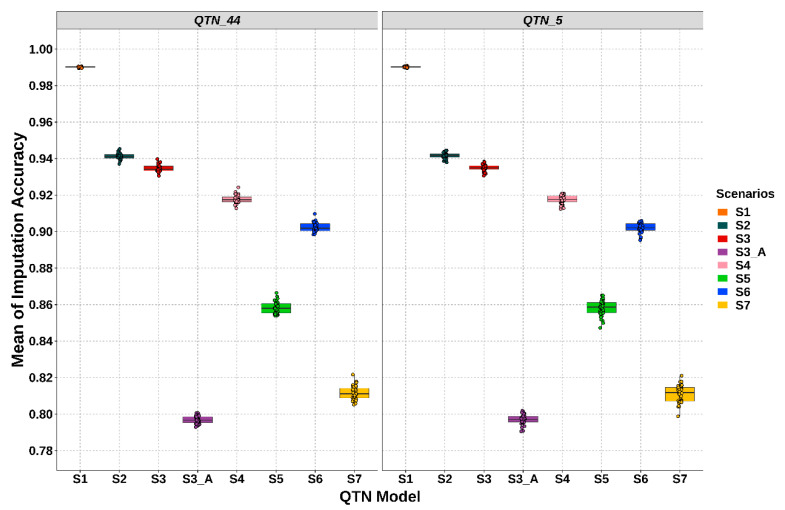
Imputation accuracy for each imputation strategy within QTN models. “S” stands for scenario. QTN_5 stands for the QTN model with 5 QTNs per chromosome and QTN_44 stands for the QTN model with 44 QTNs per chromosome. The simulated rabbit genome comprised 20 chromosomes. Each dot stands for a simulation value within each scenario.

The average of IA for the animals in the validated population (28th generation) was computed for each scenario. The results are shown in Figure 3. No difference, in terms of IA, was shown between two different QTN models. The greatest IA (0.99) was achieved in the S1. The IA decreased to 0.941 (S2) when the LD platform was used. A great decline in IA was found in S3_A (0.797) when the half validated population had imputed the whole-genome, unlike S3 (0.935). IA decreased to 0.918 when dams of the training population were genotyped with the MD platform (S4). S6 also presented an intermediate value (0.902) between all scenarios. Using LD platforms on does, IA decreases to 0.858 (S5); whereas IA declines to 0.811 with non-genotyped does (S7). In addition, lower standard deviation was obtained in S1 (0.0003), S2 (0.002) and S3 (0.002). Conversely, S7 and S5 presented the highest values of standard deviation (up to 0.005 and 0.004, respectively).

In the same way as IA, genotype yields across QTN models were higher in S1 (0.999) and S2 (0.984). The values were intermediate for S3 (0.952), S3_A (0.951) and S4 (0.945). Lower genotype yields were presented in S6 (0.899), S5 (0.893) and S7 (0.866).

### 3.3. Gemomic Prediction vs. Pedigree-Based Analyses

**Figure 4:**
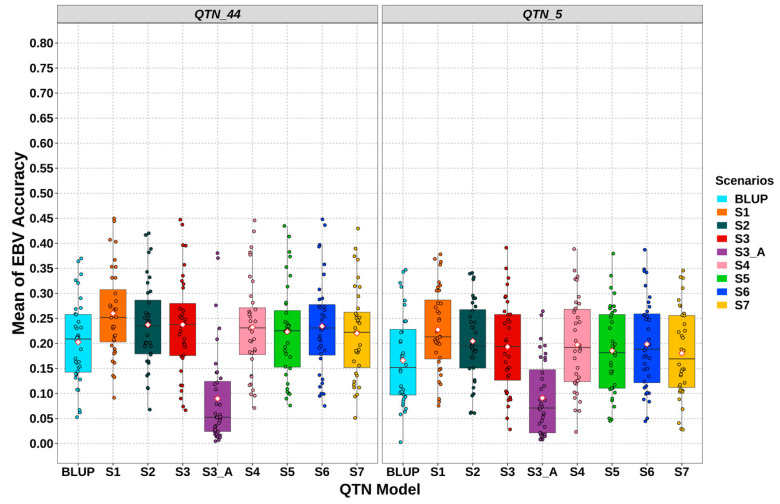
Accuracy of the estimated breeding values (EBVs) for each scenario within QTN models. “S” stands for scenario. QTN_5 stands for the QTN model with 5 QTNs per chromosome and QTN_44 stands for the QTN model with 44 QTNs per chromosome. The simulated rabbit genome comprised 20 chromosomes. Each dot stands for a simulation value within each scenario. White rhombus stands for the mean by scenario.

The accuracy of gEBVs presented, on average, a higher accuracy of prediction for QTN_44 than for QTN_5 (Figure 4). The accuracies of gEBVs of S1 were 0.26 ± 0.015 (QTN_44) and 0.228 ± 0.014 (QTN_5), representing mean ± standard error. S2 presented lower accuracy for both QTN models, 0.237 ± 0.014 (QTN_44) and 0.205 ± 0.013 (QTN_5). S3 presented similar values compared to S2, 0.237 ± 0.016 (QTN_44) and 0.193 ± 0.015 (QTN_5). S4 and S6 were very similar to S3 in QTN_44, with 0.232 ± 0.016 (S4) and 0.234 ± 0.016 (S6), and had slightly higher values than S3 in QTN_5, with 0.197 ± 0.015 (S4) and 0.199 ± 0.016 (S6). Conversely, lower accuracy values were found for S5 and S7, with 0.223 ± 0.016 (S5) and 0.22 ± 0.015 (S7) for QTN_44, and 0.185 ± 0.014 (S5) and 0.18 ± 0.015 (S7) for QTN_5. The accuracy of gEBVs drastically declined in S3_A, 0.09 ± 0.016 (QTN_44) and 0.09 ± 0.013 (QTN_5), because of low IA presented in progeny with its imputed whole-genome. The S3_A values were even worse than BLUP accuracies, which showed 0.202 ± 0.014 (QTN_44) and 0.166 ± 0.015 (QTN_5).

**Figure 5:**
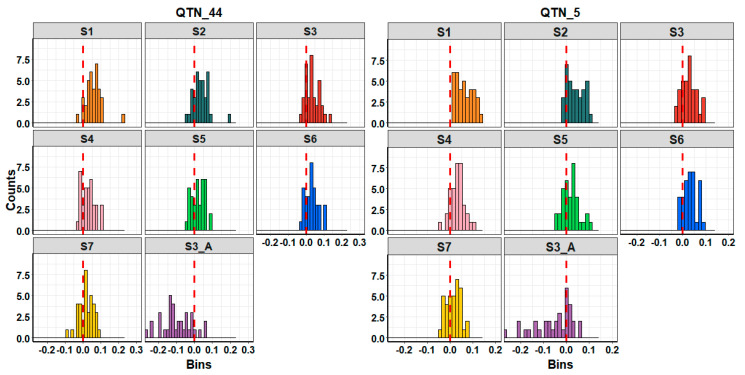
Histograms of each scenario representing the differences between accuracy from genomic predictions (ssGBLUP) versus genetic prediction (BLUP) per simulation. Right-sides of red dashed lines represent the number of simulations in which genomic selection outperformed BLUP.

The results presented a great variability on simulation-based analysis (on average S.D. of 0.08). Figure 5 shows the mathematical differences of accuracy between all genomic scenarios (S1–S7) and BLUP scenarios for each simulation. S1 presented better results than BLUP over 36 simulations for QTN_5, whilst it had better results in 95% of simulations for QTN_44. S3 outperformed in 82% of simulations, whilst S2 outperformed in 75% for QTN_5. By contrast, S3 was in the 75% of simulations better than BLUP for QTN_44, whilst S2 was in the 80%. S4 and S6 performed in a rank of 70–80%, whereas S5 and S7 performed in a rank of only 50–60%. S3_A was better than the BLUP scenario in only 25% of simulations.

The response to genomic selection and the percentages of correctly selected candidates are showed in Table 3. These parameters are strictly correlated with the accuracy of gEBVs in prolific livestock; however, they represent more pragmatic methods of comparison and dissemination for farmers. The correlations were 0.90 and 0.88 between the accuracy of gEBVs and response to genomic selection, and the first one and percentages of animals correctly selected, respectively. A response to selection of 0.105 was found when only pedigree information was used. In S1, the number of kits per generation increased by 22% with respect to BLUP. A slight decline was observed in scenarios S2, S3, S4 and S6 with a value of 0.120, 0.117, 0.114 and 0.116, respectively. S5 and S7 did not show any significant augmentation with respect to the BLUP scenario. As expected, a lower selection response than BLUP was noticed in S3_A (0.046). The percentages of correctly selected animals were similar to the trend of selection response, thus best performance was obtained in S1 with a value of 30.54%, followed by S2 and S3 with a percentage of 29.45% and 29.36%, respectively. S3 to S7 presented a range between 29.06% and 28.42%. BLUP presents a percentage of correctly selected animals of 27.81%. Even for this parameter, the values in S3_A were lower than in the BLUP scenario.

### 3.4. Genotyping Costs

**Figure 6:**
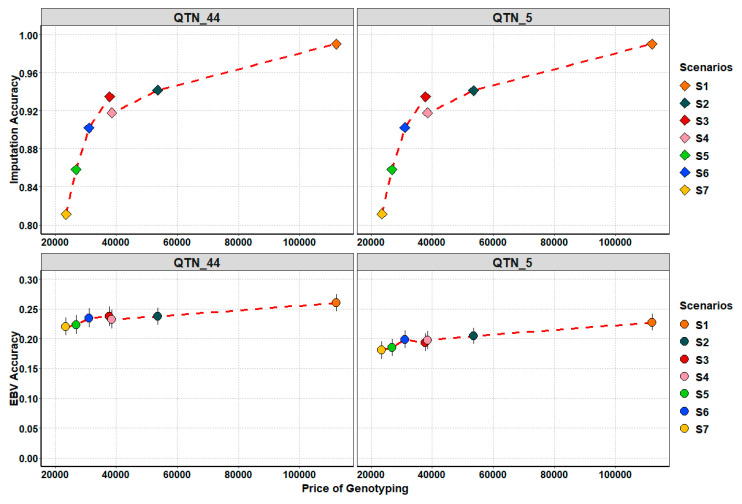
Plots of relationship between price of genotyping and imputation accuracy (top figure), and price of genotyping and EBV accuracy (bottom figure). Vertical lines are the mean ± standard error.

Figure 6 shows the relationships between the price of genotyping and IA, and the accuracy of gEBVs. As expected, the more investments, in terms of genotyping, the more IA and accuracy of gEBVs were found. However, that is not a strictly linear correlation, especially for IA. S1 showed the most expensive investment (EUR 112,000) due to progeny genotyping at the HD platform. The second ranking position was S2 with a genotyping cost of EUR 53,500. S3, S4 and S6 presented a lower cost compared to S2 from EUR 38,500 to EUR 31,000. S5 and S7 are the cheapest scenarios, with costs of EUR 26,800 and EUR 23,500, respectively.

## 4. Discussion

Our results highlight four main points for discussion: (1) imputation strategies; (2) causes that affect genomic prediction; (3) comparison with other studies; and (4) searching for trade-off: cost and genetic accuracy trends.

### 4.1. Imputation Strategies

Genotype imputation was introduced as a tool to increase detection power on association studies for linking results across studies that rely on different genotype platforms [41]. On the other hand, imputation can be a suitable tool to reduce the cost of genotyping in both plant breeding [30,42] and animal breeding programs [18,43,44]. In this case, close ancestors are typically genotyped at HD platforms, whilst progeny are genotyped at lower SNP density. When the marker position of different platforms perfectly overlaps and pedigree information is present, imputation accuracies (IAs) are usually high [29]. In our situation, in which all close ancestors are genotyped with the HD platform, this approach may not produce the same benefit as in the other species due to the prohibitive cost of HD platforms. For this reason, we examined how IAs are affected by the different genotype information of the ancestors. There are several factors influencing IA, highlighting the missing rate of LD platforms as one of the main factors [22,30]. Missing rate is the percentage of SNPs present at an HD platform that are untyped (not covered) at LD platforms. The missing rates were 70% and 99.7% for MD and LD platforms in the current study. Although the first imputation studies suggested values between 50% and 75% [29,45], the values of the current study are in line with other studies that presented very high IA for both plant [42] and animal breeding programs [18,43,46]. The missing rate can be even higher when pedigree information is available for the imputation process. AlphaImpute, which uses both genotype and pedigree information, has been demonstrated to have high imputation performance [18,29,43], even in populations with low levels of linkage disequilibrium [47]. Thus, software and missing rates seem appropriate for imputation in rabbit breeding programs.

The number of animals in the reference population, as a second factor, influences the resolution of haplotypes during the phasing process. A large number of individuals in the reference population ensures high IA in validated populations. However, it can be reduced considerably if the animals from both populations are close relatives, sharing the structure of linkage disequilibrium and haplotypes across wide chromosome segments [22,29]. S7 showed a high IA using only 70 male ancestors, which is explained by the close relationship between them, the female ancestors and progeny under ongoing selection. As expected, S1 presented the highest IA and genotype yields due to the larger number of animals in the reference population (training) and lower missing rate. In general, many studies showed that imputation using HD platforms on ancestors and MD platforms on the progenies produces high levels of IA and concordance rate in dairy cattle [46,48,49], pigs [13,18,19], poultry [12,50], sheep [20] and farmed Atlantic salmon [43]. When the SNP densities of MD (S1) platforms were reduced to LD (S2) in the validated population, IAs decreased only five percentage points. Hence, vast haplotype information keeps retrieving, using LD platforms, because of the high relatedness of rabbits. The IA results of S1 and S2 were similar to those reported in a pig imputation study, especially for Landrace and Yorkshire breeds [19]. In the current study, fewer differences in IA were found when grand-dams were genotyped at MD platforms (less than two percentage points, S3 and S4) compared to S2. This demonstrated that dams at MD platforms were enough to retrieve female haplotypes and to keep high IAs, being noticeable when S6 presented better IA values than S5. The female haplotype resolution is better when dams are genotyped at a higher SNP density than any level of SNP density on grand-dams. On the other hand, the imputation of whole genomes (S3_A) seems to not be a feasible technique for rabbit breeding programs. IA declined up to 0.797, probably due to the small number of training populations and the higher error rate of imputed SNPs associated with QTNs—very important if they have low minor allele frequency (MAF). Many animals with imputed whole-genomes presented very low IA—less than 60%. Conversely, some studies showed the benefits of strategies based on imputed whole-genomes in part of training populations [47,51] and evaluated populations using a larger number of genotyped individuals [51].

### 4.2. Causes That Affect Genomic Prediction

In this study, ssGBLUP was used as a method for genomic evaluations. This method includes a different sort of information: phenotypes, pedigree, and genotypes. Previous studies demonstrated that ssGBLUP gives potentially more accurate and less biased genomic gEBVs than multistep methods, especially in the presence of small populations and sex-limited traits [31,52].

The accuracy of the EBVs varies greatly within each scenario with respect to IA (Figure 3 and Figure 4). Conversely to the IA, which is mainly influenced by the genotyping strategies, the accuracy of gEBVs is affected by several factors. Some of these are related to the genetic architecture of the traits such as QTNs distribution and their allele frequency [22,53]. The number of SNPs in LD with these QTNs and the allele frequency of those SNPs also played an essential role in the accuracy of genomic prediction [54]. In addition, working with imputed genotypes, it is important to consider the influence of the imputation error of those SNPs and therefore IA [22,53]. In these studies, scenarios with a higher IA value also presented higher accuracy on genomic prediction and the opposite. However, a strong correlation between these two-type accuracies cannot be defined due to the high variability of gEBV accuracy within the scenarios. Additionally, with S3_A and S7, it was confirmed that low IA values (under 85%) can be deleterious for genomic prediction. For that reason, conservative thresholds for genotype imputation and quality control before genomic selection must be adopted. Furthermore, Cleveland and Hickey [18] showed that differences in gEBVs may be due to both different values of IA but also to the intrinsic genotyping structure of each scenario. There may be animals that have a marginal influence on IA but can significantly affect gEBVs.

As mentioned previously, variability in these scenarios was also caused by different allele frequencies of SNPs and QTNs. High heterogeneity of these factors was observed between simulations due to the random events that occurred during the 28th generation of mating. In some simulations, a larger number of QTNs were fixed, and in some, many SNPs were not associated with the rest of the QTNs. This would also explain the variability between simulations, and why few simulations presented a lower accuracy of gEBVs for genomic scenarios compared to BLUP, even in the scenarios with high IA as in S2 and S3.

Regarding QTN distributions, QTN_44 presented better performance than QTN_5. This trend agrees with the study of Zang et al. [53], in which ssGBLUP outperformed when the phenotype was controlled by several genes of equal effect sizes. A method that assumes unequal variances for each marker could suit for the genomic prediction of QTN_5. Modeling SNP’s effect and its variance can potentially give better results in short term selection for this simulation. Iterative ssGBLUP and/or nonlinear weight **A** can easily be implemented and can potentially lead to an increase in prediction accuracy [55,56]. Fernando et al. [57] also propose a single-step Bayesian regression in which it is possible to model the distribution of marker effects in many forms such as *t* distribution, variable selection model, and mixture distributions. Despite this, the method of estimation and of building **G** matrices was kept the same in all simulations for the sake of simplicity and for comparison with other studies.

### 4.3. Comparison with Other Studies

S1 represented the typical genotyping strategy used in all livestock species in which parents are genotyped with HD platforms and progeny are genotyped at LD platforms. As expected, S1 exhibits the best accuracies of gEBVs, and the accuracy of genomic prediction presented in S1 is close to that presented when the same candidate animals are genotyped with HD platforms; correlation is almost one in all simulations for both QTN models (data not shown). A similar correlation was found in pigs [18,58] and cattle [21,22,53], even if the proportion between HD and LD animals was different due to distinct breeding schemes present in these species. Previous imputation studies conducted in commercial pig breeding programs are a good comparison method due to a similar mating system, although the number of animals is lower in rabbits. Scenarios comparable to those present in this study have been reported in Cleveland and Hickey [18]. A similar sharp drop in IA and the accuracy of gEBVs was observed when animals of validated populations were genotyped with MD to LD platforms. In addition, an analogous decline in gEBVs was observed when grand-dams and dams were genotyped with LD or MD, although in both studies IA was quite high for these scenarios. However, Cleveland and Hickey [18] demonstrated that genotyping at HD platforms for animals that are not related with LD animals has little impact concerning IA, but it can significantly affect the accuracy of gEBVs. Nevertheless, this scenario was not included in our study because the limited number of animals would not guarantee the same gap of accuracy presented in pigs. The same consideration made on Grossi et al. [19] can be also made for our study; genotyping reference animals with the LD platform can ensure good accuracy levels of IA and gEBVs when parents are genotyped with HD, if not low levels of prediction have been observed, i.e., S5 or S7.

### 4.4. Searching for Trade-Off: Cost and Genetic Accuracy Trends

Cost evaluations are usually performed by comparing imputation strategies against an idealistic genomic selection in which all candidate animals are genotyped at HD platforms. Under this condition, imputation strategies are always a cost-effective technique [18,42,43]. Here, we show only genotyping costs to evaluate investment for moving from traditional genetic evaluation (BLUP) to genomic selection (ssGBLUP). Imputation was further demonstrated as a reliable tool for reducing genotyping investment, with an accuracy higher than BLUP in the majority of cases.

However, some scenarios present a cost-benefit inefficiency when compared with the other scenarios. Clearly, S3_A cannot be taken as an option due to the low performance of prediction—even worse than BLUP. Results from this scenario suggest that a method based on high thresholds of individual-specific IA must be considered, especially when genomic characterization is available [22], and for strategies with non-genotyped animals [51]. Using animals with imputed whole-genomes for genomic selection is still a challenge due to the imputation error rate [51,59]. In this sense, high error rates of imputed SNPs associated with QTNs are clear in S3_A, presenting 75% of simulations lower than BLUP scenarios. BLUPF90 reported genotypes duplicated for S3_A, as *AlphaImpute* copied whole-genomes of full-sibs for some progenies.

The same reason can be applied to S7 and S5 as, even if lower genotype investment was present in these scenarios, these strategies cannot be considered one of the best scenarios because the number of simulations in which genomic selection outperformed BLUP is limited—about half of the cases. Conversely, S3, S6, and S4 presented a considerable increase in accuracy and response to selection with a marginal increment investment.

The opposite situation was presented in S1, and even if a significant reduction in accuracy was observed switching from MD platforms to LD platforms (S2), the big drop in price observed can potentially justify this decline in accuracy. No significant differences in terms of percentage of correctly selected animals and response to selection were observed between S2 with S3 and S6, thus these two scenarios are preferable over S2 due to the lower cost. In addition, S4 can be discarded among the best scenarios, as it presents a lower selection response than S6 and S3 but at a higher price. In conclusion, the best scenarios can be identified as S3 and S6. Genotyping only half of the animals per litter (S3) is an effective strategy to reduce the impact of the cost of genotyping, especially in prolific species such as rabbit and pig. This approach is commonly adopted in the pig industry selection scheme [15,17,18]. On the other hand, S6 demonstrated that genotyping grand-dams (S4) leads to a negligible difference in accuracies when sires and dams are genotyped, with S6 being cheaper than S3 (+ EUR 6750). Thus, we considered S6 as the strategy with the best trade-off for implementing genomic selection in rabbits.

## 5. Conclusions

Imputation strategies are feasible in rabbit breeding programs as in other species, as IAs were high, particularly in scenarios with parents’ genotype at HD platforms. Furthermore, the slight positive correlation between IA and the accuracy of gEBVs was also demonstrated, and scenarios with IA also have a high gEBV accuracy. The results of the response to genomic selection and the percentage of correctly selected candidates are in the same line as genetic accuracy values. Despite this, gEBV accuracy showed great variance among simulations. Therefore, we must be cautious with the results from simulated data as they are conditioned to several factors of the small reference population (e.g., size, relationships between individuals, inbreeding level, and update). Another comparison between genomic selection and BLUP could be made by enlarging the reference population using dams of the nucleus farms and the multipliers, or even crossbreeding from commercial farms. In conclusion, the adoption of imputation strategies can be an effective strategy for drastically reducing the genotyping cost in rabbits, maintaining an accuracy slightly lower than that with all HD animals. Hence, the best trade-off scenario can be identified in S6; although, as previously stated, these results may change under different conditions.

## Figures and Tables

**Table 1:** Structure of imputation strategies and number of genotyped animals for implementing genomic selection in rabbits.

Imputation Strategy	Training Populations				For Imputation	Validated Population (Genomic Prediction)
	26th Generation		27th Generation		28th Generation	
	Grand-Dams (150)	Grand-Sires (35)	Dams (150)	Sires (35)	Progeny (1500)	Progeny (1500)
S1	HD	HD	HD	HD	MD	*i-*HD
S2	HD	HD	HD	HD	LD	*i-*HD
S3	MD	HD	HD	HD	½ LD	½ *i-*HD + ½ NG
S4	MD	HD	MD	HD	LD	*i-*HD
S5	LD	HD	LD	HD	LD	*i-*HD
S6	NG	HD	MD	HD	LD	*i-*HD
S7	NG	HD	NG	HD	LD	*i-*HD
S3_A	MD	HD	HD	HD	½ LD	½ *i-*HD + ½ *i-*WG

The “S” stands for scenarios. Animals of every scenario were genotyped according to different Single Nucleotide Polymorphism (SNP)-density platforms. HD: high SNP-density platform (200,000 SNPs), MD: medium SNP-density platform (6000 SNPs), LD: low SNP-density platform (600 SNPs). *i-*HD: animals with imputed genotypes to high SNP-density. *i-*WG: animals with imputed whole-genome. NG: non-genotyped animals.

**Table 2:** Number of data used in the genomic analyses per generation.

Generation	Pedigree	Phenotypic ^1^	Genomic ^2^
23th	300	150	0:0
24th	300	150	0:0
25th	300	150	0:0
26th	300	150	35:150
27th	300	150	35:150
28th	1500	0	0:1500

^1^ Selected does are 47% of total females (150). Each female contributes to the progenies with the same proportion of males and females (1:1). ^2^ Number of males: females with genotypes in each generation. The number varies according to the imputation strategy given standard *BLUPF90* parameters of quality control.

**Table 3:** Response to genomic selection and percentages of correctly selected animals across QTN models for each scenario.

Scenario	Selection Response ^1^	SE-1 ^2^	Percentage of ACS ^3^	SE-2 ^4^
BLUP	0.105	0.007	27.81	0.56
S1	0.129	0.007	30.54	0.56
S2	0.120	0.007	29.45	0.54
S3	0.117	0.008	29.36	0.60
S3_A	0.046	0.008	23.62	0.65
S4	0.114	0.007	28.93	0.54
S5	0.108	0.007	28.42	0.53
S6	0.116	0.007	29.06	0.55
S7	0.109	0.007	28.26	0.56

^1^ Mean of response to genomic selection (kits). ^2^ Standard error of response to genomic selection. ^3^ Percentage of animals correctly selected. ^4^ Standard error of percentage of animals correctly selected.

## Data Availability

The databases used and analyzed in the current study are available from The Figshare Repository. Some scripts are also included in the repository. The link addresses are https://doi.org/10.6084/m9.figshare.13350443 or https://figshare.com/s/2591beabd3ef5bc6d220 (accessed on 9 March 2021).

## Supplementary Materials

The following are available online at https://www.mdpi.com/2076-2615/11/3/803/s1, Table S1: Parameters and info to run simulations in *AlphaSim* and *MaCS*.
