# Supplementary material for: Genotype Imputation to Improve the Cost-Efficiency of Genomic Selection in Rabbits
Source: Animals (Basel). 2021 Mar 13;11(3):803. doi: 10.3390/ani11030803 (PMC8000098; doi:10.3390/ani11030803)
Supplement: Supplementary file 1 [file animals-11-00803-s001.pdf]

Table 1. Parameters and info to run simulations in *AlphaSim* and *MaCS.*

**1. Pedigree and Animal Breeding Options**				
**Generation**	**Number of Animals**			**Selection Method**
	**Total Population**	**Sire**	**Dam**	
1st–3rd	300	150	150	Random Mating
4th	400	109	153	Random Mating
5th	324	77	138	Random Mating
6th–25th	300	35	70	Pedigree BLUP
26th	300	35	70	GBLUP
27th	300	35	150 *	GBLUP
28th	3000	----	----	-----
**2. Chromosomes**				
Population History *MaCS*			Internal Rabbit	
Chromosome Length Bases *MaCS*			External, 124.43E+06	
Number Of Chromosomes			20	
Number Of Haplotypes			2000	
**3. SNP Chips Features**				
Number Of SNP Chips			3	
**Number of SNP Per Chip Per Chromosome**		**1 (HD)**	**2 (MD)**	**3 (LD)**
		10000	300	30
Min Allele Frequency For SNP		0.40	0.40	0.40
Max Allele Frequency For SNP		0.50	0.50	0.50
SNP Chip Includes QTN			Random	
SNP Chips Are Nested			Yes	
Id Of Chip Used For Selection			1 (HD)	
**4. QTN Features**				
Number Of QTN Per Chromosome Gamma Distribution			5 or 44	
Shape And Scale Of Gamma Dist. For Model 3 And 4			0.60, 0.80	
Dominance Degree Mean And Variance			0.0, 0.0	
QTN Clusters			Off	
**5. Selection Process**				
Selection Based On QTN			Unrestricted	
Distribution Of QTN Of Selection Traits			Gamma	
**Selection Phenotyping Strategy (SPS)**				
		**SPS**	**Generation**	**Selection Method**
		All Females	6th–25th	Pedigree BLUP
		Random Phenotypes	26th–27th	GBLUP
**Features of Random Phenotypes**			**Generation**	**Item**
**First And Last Training Gen For Random Phenotypes Strat.**			26th	25, 25
			27th	25, 26
**Training Set Size For Random Phenotypes Strat.**			26th	150
			27th	150, 150
Use Training Data From Generation			25th	
Generations Of Pedigree Prior To Training			5th	
**4. Traits**				
Number Of Traits			1	
Index Weights			1	
Trait Heritability			0.113	
Trait Genetic Variance			0.6753	
Genetic Correlation Matrix			1.0	
Residual Correlation Matrix			1.0	

* Total number of evaluated dams (does) used with their progenies at 28th generation for genomic prediction.
HD: high SNP-density; MD: medium SNP-density; LD: low SNP-density.
